# Genetic engineering of *Synechocystis* PCC6803 for the photoautotrophic production of the sweetener erythritol

**DOI:** 10.1186/s12934-016-0458-y

**Published:** 2016-04-08

**Authors:** Aniek D. van der Woude, Ruth Perez Gallego, Angie Vreugdenhil, Vinod Puthan Veetil, Tania Chroumpi, Klaas J. Hellingwerf

**Affiliations:** Photanol BV, Science Park 408, 1098XH Amsterdam, The Netherlands

**Keywords:** Cyanobacteria, Metabolic engineering, Sweetener, Erythritol, Calvin cycle, Erythrose, Erythrose-4-phosphate

## Abstract

**Background:**

Erythritol is a polyol that is used in the food and beverage industry. Due to its non-caloric and non-cariogenic properties, the popularity of this sweetener is increasing. Large scale production of erythritol is currently based on conversion of glucose by selected fungi. In this study, we describe a biotechnological process to produce erythritol from light and CO_2_, using engineered *Synechocystis* sp. PCC6803.

**Methods:**

By functionally expressing codon-optimized genes encoding the erythrose-4-phosphate phosphatase TM1254 and the erythrose reductase Gcy1p, or GLD1, this cyanobacterium can directly convert the Calvin cycle intermediate erythrose-4-phosphate into erythritol via a two-step process and release the polyol sugar in the extracellular medium. Further modifications targeted enzyme expression and pathway intermediates.

**Conclusions:**

After several optimization steps, the best strain, SEP024, produced up to 2.1 mM (256 mg/l) erythritol, excreted in the medium.

**Electronic supplementary material:**

The online version of this article (doi:10.1186/s12934-016-0458-y) contains supplementary material, which is available to authorized users.

## Background

Erythritol is a natural 4-carbon sugar polyol that is commonly used in the food and pharmaceutical industries. It is an increasingly popular sweetener, as it has ~60 % of the sweetness of sucrose and is almost non-caloric (i.e. not metabolized by human enzymes) as well as non-cariogenic [1–3]. Erythritol occurs naturally as a metabolite in several fruits, fungi and bacteria [2, 4]. Its chemical synthesis is complicated and commercial production of this polyol is therefore based on fermentation, mostly using osmophylic fungi such as *Torula* sp. and *Moniliella pollinis* [3]. These species naturally produce erythritol (as an osmoprotectant; see further below) and have been optimized for industrial production by adjusting growth media and growth conditions only. The highest yield of erythritol on glucose reported is 61 % [5]. Owing to its increasing demand in the food industry, there is a need for optimized production methods for erythritol.

Several biotechnological strategies have been applied to divert the production of bio-commodities away from glucose, as this substrate so far has mostly served as the feedstock. The most sustainable approach is turning out to be direct photosynthesis-based production, which has been demonstrated using various cyanobacteria as the producing host organism. By expression of a specific (set of) heterologous gene(s) encoding metabolic enzymes, jointly forming a product-forming pathway, and expressed in a particular cyanobacterium such as *Synechocystis* PCC6803 (hereafter, *Synechocystis*), proof of principle has been provided for production of numerous compounds like butanediol, ethanol, ethylene, isobutanol, lactate and various terpenoids [6–8]. Here, we show successful production of erythritol using a specifically engineered *Synechocystis* strain.

**Fig. 1:**
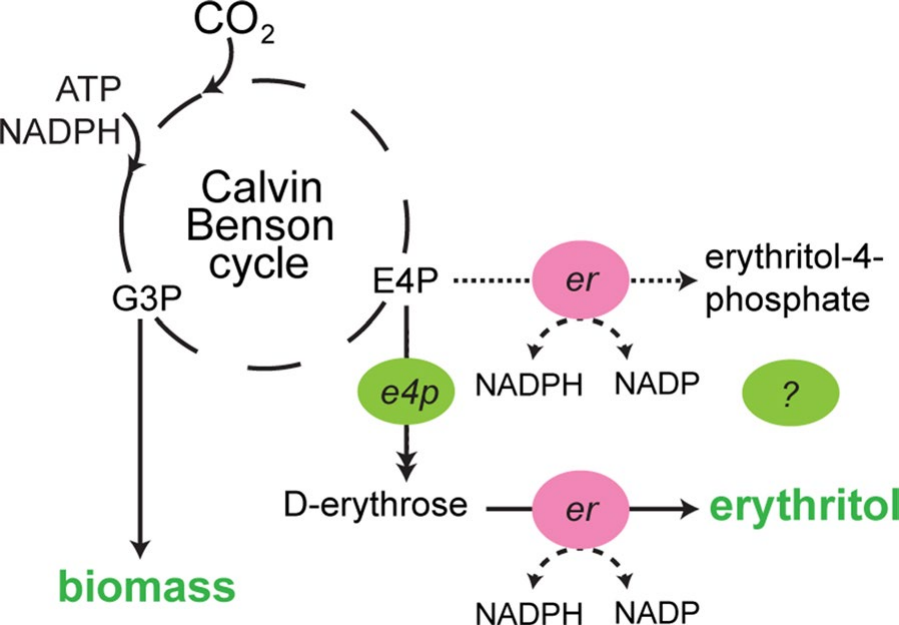
Schematic representation of engineered erythritol production in *Synechocystis*. Erythritol can be produced with introduction of two genes, encoding an erythrose-4-phosphatase (e4P) and an erythrose reductase (er), respectively. The ER is also able to reduce erythrose-4-phosphate, as indicated with the dashed line, but whether a phosphatase is present to further process the product of this reaction to erythritol is unknown

Erythritol can be formed in a two-step pathway from the pentose phosphate pathway intermediate d-erythrose-4-phosphate. The pathway of erythritol formation has been best studied in fungi, where erythritol can serve as an osmoprotectant. When encountering salt or osmotic stress, these organisms produce compatible solutes. Although glycerol is the best-known osmoprotectant, erythritol is also used to protect cells against osmotic stress. The pathway proceeds via dephosphorylation of d-erythrose-4-phosphate (E4P) to d-erythrose, followed by reduction to erythritol (Fig. 1). Several erythrose reductases, derived from *e.g.Candida magnoliae, Trichosporonoides megachiliensis,Saccharomyces cerevisiae* and *Trichoderma reesei* have been identified, purified and characterized [9–12]. Each of these reductases depends on NADPH as the redox co-factor, which is also the primary reductant available under photoautotrophic conditions in cyanobacteria [13]. The (catabolic) pathway for erythritol production, and its physiological function, is supposedly different in bacteria, such as described for *Oenococcus oeni*, where E4P is first reduced to erythritol-4-phosphate and then dephosphorylated. Here, erythritol presumably functions as a redox sink during glucose fermentation [14]. However, the enzymes involved in this pathway are still unknown [15].

In this study, several different erythrose-4-phosphatases and erythrose reductases were introduced into *Synechocystis* to demonstrate erythritol production, tapping off directly from E4P, a key intermediate of the CO_2_-fixing Calvin Benson Bassham cycle (Fig. 1). These results demonstrate the feasibility of direct photosynthesis-based production of erythritol using cyanobacteria.

## Methods

### Bacterial strains and growth conditions

*Escherichia coli* strains XL-1 blue (Stratagene) or EPI400 (Epicentre biotechnologies) were used for plasmid amplification and manipulation, grown at 37 °C in Lysogeny Broth (LB) or on LB agar. *Synechocystis* sp. PCC6803 (glucose tolerant, obtained from D. Bhaya, Stanford University, USA) was routinely grown at 30 °C in liquid BG-11 medium (Sigma-Aldrich), supplemented with 10 mM TES-KOH (pH 8) or 25 mM CAPSO (pH 9) and appropriate antibiotics, and incubated with shaking at 120 rpm (Innova 43, New Brunswick Scientific) under moderate intensity white-light illumination (~35 μE/m^2^/s) or under high intensity illumination (~100 μE/m^2^/s; combining 90 % red and 10 % blue light) to optimize growth rate. Growth of *Synechocystis* strains was monitored by following OD_730_ (Spectrophotometer Lightwave II, Biochrom) at selected time intervals. BG-11 agar plates were supplemented with 10 mM TES-KOH (pH = 8), 0.3 % (w/v) sodium thiosulfate and 5 mM glucose. When appropriate, the following antibiotics were used: ampicillin (100 µg/ml), kanamycin (20 or 50 µg/ml, for *Synechocystis* and *E. coli*, respectively), spectinomycin (25 µg/ml), streptomycin (10 µg/ml), and chloramphenicol (20 µg/ml).

Natural transformation for genomic integration of exogenous genes in *Synechocystis* was performed as described previously [16], using plates with increasing concentrations of antibiotic for growing the transformants to drive segregation. Conjugation of RSF1010-based plasmids from *E. coli* XL-1 to *Synechocystis* was performed by tri-parental mating using *E. coli* J53 (pRP4) as the helper strain, essentially as described in [17]. Correct insertion of the genes and full segregation, as well as insertion of conjugation plasmids, were verified by colony PCR with specific primers (Table S1) and *Taq* DNA polymerase (Thermo Scientific), and subsequent sequencing of the amplified fragment.

### Molecular cloning

Codon-optimized sequences encoding the heterologous TM1254, ErCm, Gcy1p, YidA, GLD1, ALR1 and Pc20g15580 enzyme were synthesized and directly inserted into pHKH001 [16], pUC57 or PCC by Genscript (Piscaway, NJ, USA), flanked by a P*trc1* promoter, the transcriptional terminator BBa_B0014 and Biobrick compatible restriction sites. Codon optimization was performed using the OPTIMIZER application and the codon usage table of the cyanobase website (http://www.kazusa.or.jp/codon/cgi-bin/showcodon.cgi?species=1148). Unwanted restriction sites present in the coding sequences were removed using the same OPTIMIZER application [18]. Further specific details on plasmids used in this study are listed in Additional file 1: Table S2. PCR reactions for cloning procedures and amplification prior to sequencing were carried out using the *Pfu* DNA polymerase (Thermo Scientific) or Velocity DNA polymerase (Bioline).

For the construction of pVPV003, we replaced the *erCm* gene of pVPV002 with *gcy1p* by digestion of pVPV001 and pVPV002 with NheI/PstI or AvrII/PstI, respectively, followed by ligation. Moreover, the *tm1254* gene plus *trc1* promoter was taken from pVPV002 by EcoRI/AvrII digestion and inserted into EcoRI/XbaI digested, and RSF1010-based plasmid pJBS1250 [19]). The resulting vector, pVZ-TM1254 was used as a basis to create plasmids pAW029 and pAW030. To this end pVZ-TM1254 was digested with SpeI/PstI, and ligated with XbaI/PstI inserts taken from pHKH-ErCm and sVPV001, respectively. pHKH-ErCm was constructed earlier by removing the *tm1254* gene with NheI/AvrII and subsequent ligation. For expression in *E. coli* the *tm1254, erCm* and *gcy1p* genes were amplified with specific anchoring primers, using pVPV001 and pVPV002 as the template. Subsequently, both the *E.coli* expression vector pQE30 (Qiagen), as well as the respective PCR products, were digested with BamHI/HindIII and ligated.

The gene encoding the Sll1524 phosphatase was amplified from the *Synechocystis* genome with specific primers and ligated into the pHKH001 vector, together with a *trc1* promoter and the BB0014 terminator. Next, the homologous regions of the pHKH-sll1524 vector were successively replaced by longer fragments, and amplified with specific anchoring primers from the *Synechocystis* genome, finally resulting in pHeKHe-sll1524. This vector was digested with XbaI-PstI to replace the phosphatase gene cassette with either *tm1254* or *yidA*, obtained with the same restriction enzymes from pVZ-TM1254 or pUC57-YidA, respectively. Next, these three phosphatase containing vectors were opened by SpeI-PstI digestion to allow for insertion of the XbaI-PstI digested gene cassettes encoding the erythrose reductases Gcy1p, ALR1, GLD1 or Pc20g15580. This resulted in the vectors pEP001 to pEP012 (excluding pEP011; see Additional file 1: Table S2).

Next, we constructed several RSF1010-based conjugation plasmids for the introduction of heterologous genes into *Synechocystis*. First, we added the XbaI-PstI digested *gld1* gene cassette to the pVZ-TM1254 plasmid that was digested with SpeI-PstI, resulting in pVZ-TM1254-GLD1. Subsequently, we replaced the kanamycin resistance cassette of pVZ-TM1254 with an omega cassette. For this, we amplified the omega cassette with MluI-anchored primers, using pDF-lac [20] as the template. This product was then inserted into pVZ-TM1254-GLD1 by MluI digestion, resulting in pAVO-TM1254-GLD1. This vector was used to make strain SEP013.

To vary the promoter sequence of the phosphatases, both TM1254 and YidA were cloned using NdeI-BamHI digestion into a pAVO-cYFP vector, digested with the same enzymes. This latter vector was constructed by inserting into the pAVO backbone plasmid (through EcoRI-BamHI digestion) both the *cpcBA* promoter and the His10-labelled YFP from the pAQ1ExPcpcBA::eYFP SpR [21], kindly provided by D. Bryant. After introduction of the phosphatases, this resulted in the vectors pAVO-cTM1254 and pAVO-cYidA.

Finally, since we experienced serious cloning difficulties with the RSF1010-derived plasmids, we introduced the Y25F mutation in the *mobA* gene of pAVO-cTM1254 to reduce its ability for auto-mobilization and improve its digestibility, as described by [22]. The resulting vector pAVO+-cTM1254 was then used to digest with SpeI-PstI and insert the XbaI-PstI-digested *gld1* gene cassette. The resulting vector is pEP021.

For overexpression of the transketolase and phosphoketolase, we did not manage to clone the genes into the desired vectors, likely due to toxicity issues in *E. coli.* Therefore, we instead used the fusion PCR method to transfer these genes into the cyanobacterial genome. The transketolase that we selected is the *SynPCC7002_A1022* gene, amplified from *Synechococcus* PCC7002. Interestingly, we were unable to detect a phosphoketolase in this species and therefore chose to amplify this gene from the genome of *Synechococcus elongatus* PCC7942, gene *SynPCC7942_2080.* These PCR fragments were combined with two amplicons of the phaAHCmH vector [23] that contained a gene with a C-terminal His-tag behind a t*rc1* or *psbA2* promoter in front of the chloramphenicol resistance cassette, in a single fusion PCR reaction with 30 bp overlapping regions. The resulting fragments were used directly for transformation of *Synechocystis.*

### Preparation of *Synechocystis* lysates for measurement of intracellular metabolites

Lysates for the analysis of intracellular metabolites were prepared essentially as described by [24], using pelleted 10 ml *Synechocystis* cultures with an OD_730_ of ~1.0 and ~6.0, to represent exponential and stationary growth phase, respectively. In short, samples were dissolved in 5 ml 100 % ethanol and boiled at 65 °C for 4 h. Supernatants were collected, dried under a N_2_ stream and dissolved in 500 μl of de-ionized water. Before HPLC analysis samples were treated with perchloric acid and KOH (as described below). To estimate the intracellular concentration of metabolites, we assumed that 1 ml of culture at OD_730_ = 1 contains 7 × 10^7^ cells and that each cell has a diameter of 2 μm. With these data, we could calculate to what extent the intracellular metabolites were diluted upon cell lysis.

### Erythritol quantification by HPLC

To determine extracellular erythritol concentrations, supernatant samples of cultures were subjected to HPLC analysis. HPLC samples were either directly filtered or prepared by treating 500 μl supernatant sample with 50 μl of 35 % (v/v) perchloric acid (Merck), incubation on ice for 10 min and subsequent addition of 27 μl of 7 M KOH (Merck). After vortexing, the precipitate was removed by centrifugation for 5 min at 12,000 rpm and subsequent filtering (Sartorius Stedin Biotech, minisart SRP4, 0.45 μm). Separation of organic acids was achieved by application of a 20 μl aliquot on a Rezex ROA-Organic Acid H^+^ (8 %) HPLC column (Phenomenex), coupled to a refractive index detector (Jasco, RI-1530), using a flow of 0.5 ml/min and a column temperature of 85 °C. The concentration was determined by comparison of its peak size with known amounts of meso-erythritol (Sigma-Aldrich) and d-erythrose (Sigma-Aldrich).

### Preparation of *Synechocystis* soluble lysates and enzymatic activity assays

Soluble lysates of *Synechocystis* were obtained after harvesting a culture during late-exponential growth with an OD_730_ of ~1.0 (Spectrophotometer Lightwave II, Biochrom) by centrifugation (10 min at 4000 rpm) at 4 °C. The resulting cell pellets were dissolved in pre-chilled 100 mM phosphate buffer (pH = 7.6) with 10 % glycerol and disrupted with 100 μm glass beads (Sigma) using a Precellys^®^24 bead beater (Bertin technologies). After removal of cell debris by centrifugation (30 min at 14,000 rpm) at 4 °C, the protein concentration of these samples was measured using the BCA protein assay (Pierce).

Enzymatic activity assays for the ER enzyme were essentially performed as described in [25], using a 50 mM phosphate buffer pH = 7.6, containing 300 μM NADPH, and starting the reaction with the addition of 20 mM d-erythrose. NADPH consumption, measured at 340 nm and at 30 °C, was recorded as a measure for reductase activity. Activities were corrected for the endogenous, substrate (i.e. erythrose) independent, rate of NADPH consumption.

### SDS-PAGE

Samples were dissolved in protein solubilisation buffer (50 mM Tris–HCl pH 6.8, 100 mM dithiotreitol, 50 mM EDTA, 2 % (w/v) sodium dodecylsulphate, 10 % (v/v) glycerol) and incubated at 95 °C prior to SDS-PAGE analysis. Proteins were separated by SDS-PAGE and stained with Coomassie Brilliant Blue (CBB) G-250, *i.e.* PageBlue Staining solution (Thermo scientific), according to manufacturer’s protocol, or transferred to nitrocellulose membranes for Western blotting. These membranes were incubated with mouse monoclonal antibodies directed against the histidine epitope (11922416001; Roche Applied Science). Secondary horseradish peroxidase-conjugated goat anti-mouse IgGs were detected with ECL (Pierce).

## Results and discussion

### Extracellular erythritol is not consumed by *Synechocystis* and exhibits low toxicity

**Fig. 2:**
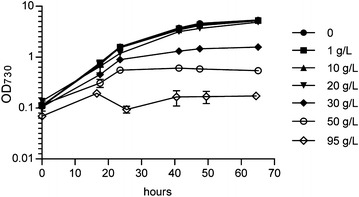
Effect of extracellular erythritol on growth of *Synechocystis.* Growth curve of wildtype *Synechocystis,* cultured in the presence of 0, 1, 10, 20, 30, 50 or 95 g/l *meso*-erythritol. Cells were grown under high intensity *red*/*blue*-light illumination (~70 μE/m^2^/s) in BG-11 supplemented with 10 mM TES-KOH (pH = 8) and 50 mM NaHCO_3_ at 30 °C. *Error bars* represent SD (n = 2)

To evaluate the effects of the addition of extracellular erythritol on the growth of *Synechocystis*, cells were inoculated in the absence and presence of 1, 10, 20, 30, 50 and 95 g/l erythritol in BG-11 medium and with illumination optimized for rapid growth (see “Methods” section). Up to a concentration of 20 g/l (*i.e.* 164 mM) no significant effect on growth (neither rate nor yield) was detected under the high-light conditions (Fig. 2). Therefore, it can be concluded that concentrations of erythritol up to this level are not toxic for growth of *Synechocystis.* In the presence of 30, 50 and 95 g/l erythritol, growth rates during the first 24 h decreased to roughly 80, 60 and 10 % of the rate of the wild-type culture, respectively, and after that growth was almost completely inhibited. At these very high concentrations it seems likely that erythritol causes osmotic stress to the cells similar to sorbitol [26]. These results indicate that erythritol is a suitable target compound for production by an engineered cyanobacterium, as long as titers will not reach much higher levels than 160 mM. Moreover, the supernatants of these cultures were analyzed before and at the end of the assay for their erythritol concentration, using HPLC analysis. The detected erythritol levels did not change over the course of the experiment (results not shown) and we therefore conclude that *Synechocystis* does not significantly consume extracellular erythritol.

### Strain construction for the production of erythritol

For the production of erythritol by *Synechocystis* PCC 6803, reductase genes from yeast- and fungal catabolic pathways were chosen for expression in the cyanobacterium. Different erythrose reductases have been described in literature and, based on their enzyme-catalytic properties, we initially selected the reductase CmER of *Candida magnolia* JH110 [11] and the reductase Gcy1p, derived from *Saccharomyces cerevisiae* S288c [10]. In contrast to the reductases, available knowledge on the enzymology of the phosphatases is very limited and the responsible enzyme in the fungal catabolic pathway has not been annotated. As the first candidate, a HAD-like phosphatase, TM1254, derived from *Thermotoga maritima* MSB8, was chosen, which was detected in a large screen for phosphatase activity as being relatively specific for erythrose-4-phosphate [27]. All these genes were codon-optimized for expression in *Synechocystis* and obtained through chemical synthesis (see “Methods” section). Next, *tm1254* was cloned in an operon together with *cmER* or *gcy1p*, which was expressed with a *trc1* promoter, after integration into the *Synechocystis* genome together with a kanamycin resistance cassette, at the neutral site *slr0168.* After segregation, this resulted in strains SVPV2 and SVPV3, respectively. However, even after long-term growth and multiple erythritol-production assays, no production of erythritol was detected for these strains (results not shown).

**Fig. 3:**
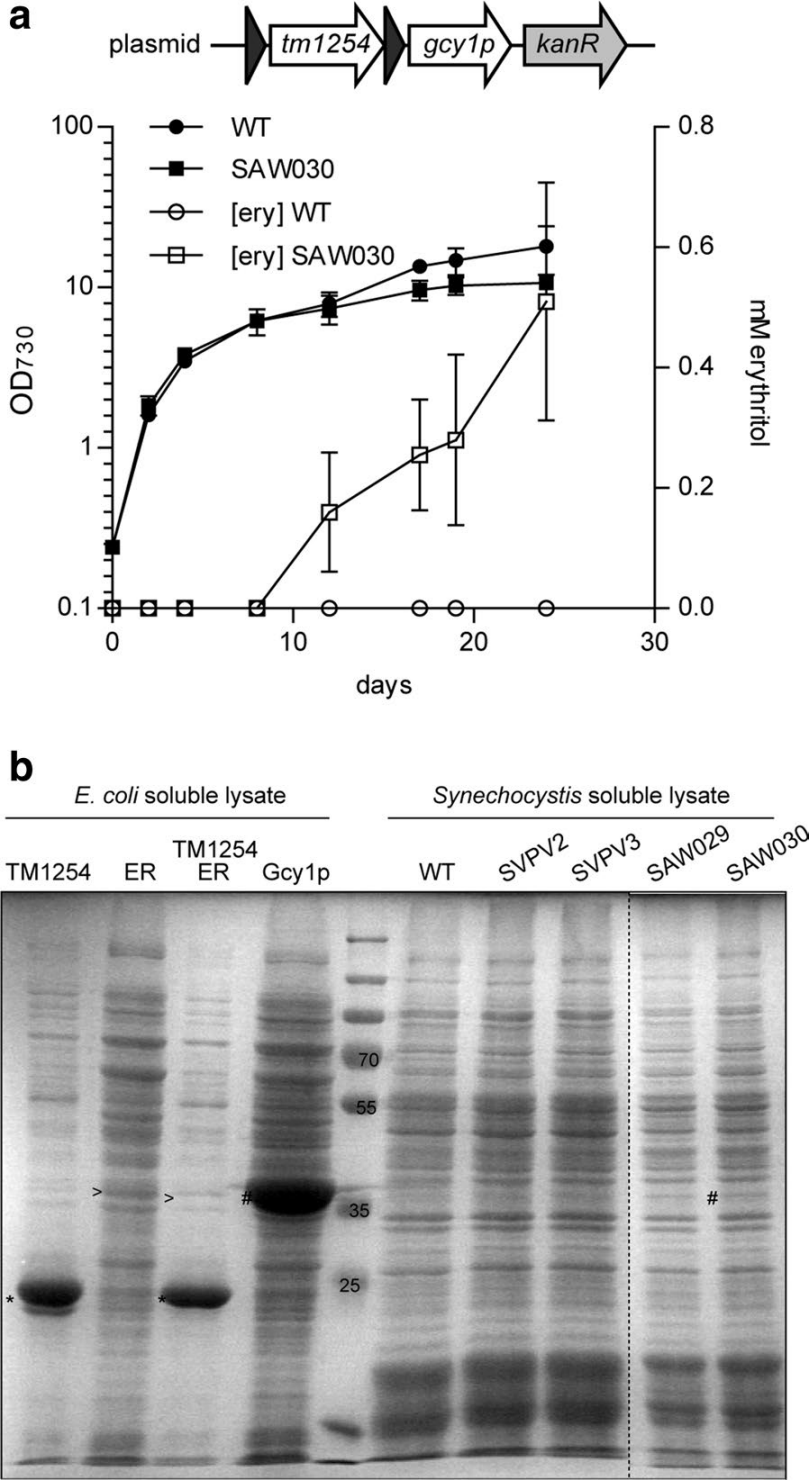
Photoautotrophic production of erythritol by an engineered cyanobacterium. **a** Growth and extracellular erythritol production of wildtype *Synechocystis* (WT) and mutant SAW030. *Solid symbols* represent OD_730_, whereas *open symbols* depict the erythritol concentration. *Error bars* represent SD (n = 2). **b** CBB-stained SDS-PAGE showing *Synechocystis* soluble lysates of wildtype and the different erythritol-producing mutants and *E. coli* soluble lysates with overexpressed TM1254 (indicated with *), ErCm (ER >) and Gcy1p (#), respectively

Additionally, we introduced the *tm1254* gene and *cmER* or *gcy1p* as individual cassettes, each expressed from their own *trc1* promoter, into the RSF1010-derived plasmid pVZ322. Introducing these plasmids into *Synechocystis* via conjugation resulted in strains SAW029 and SAW030, respectively. These strains were also tested for erythritol production in a long-term growth experiment. This experiment showed that only strain SAW030, i.e. expressing *tm1254* and *gcy1p*, produced measurable amounts of erythritol. Growth of SAW030 is highly comparable to that of the corresponding wild type *Synechocystis* strain (Fig. 3a). This figure shows an experiment (using each strain in duplicate) to measure growth and the production of erythritol in the culture medium/supernatant of the cells. Erythritol concentrations were measured by HPLC. Interestingly, no extracellular erythritol can be detected during exponential growth of the culture. Only from day 12 onwards, when the culture has reached the stationary phase, extracellular erythritol is produced to detectable levels. Significantly, the related intermediate d-erythrose was not detected. However, coinciding with the production of erythritol we also measured increasing levels of a product at a different retention time, which we identified as glycerol. The maximal erythritol levels that have been observed for SAW030 are 0.71 mM after 35 days of growth (Table 1). Nevertheless, in this mutant the glycerol levels produced actually were even higher, namely 2.3 mM (data not shown).

**Table 1:** List of engineered *Synechocystis* strains for erythritol production

	Genotype	Parent strain	Segregated	Highest measured concentration erythritol [#days—OD730]
VPV2	*slr0168::Ptrc:tm1254:cmER*-*kanR*	WT	Yes	No erythritol [24d–19.2]
VPV3	*slr0168::Ptrc:tm1254:gcy1p*-*kanR*	WT	Yes	No erythritol [24d–9.2]
SAW029	*pVZ*-*Ptrc:tm1254*-*Ptrc:cmER*-*kanR*	WT	Plasmid	No erythritol [24d–8.8]
SAW030	*pVZ*-*Ptrc:tm1254*-*Ptrc:gcy1p*-*kanR*	WT	Plasmid	0.71 mM [35d–12.3]
SEP001	*slr0168::Ptrc:tm1254*-*Ptrc:gcy1p*-*kanR*	WT	Yes	0.1 mM [24d–8.0]
SEP002	*slr0168::Ptrc:yidA*-*Ptrc:gcy1p*-*kanR*	WT	Yes	No erythritol [24d–6.5]
SEP003	*slr0168::Ptrc:sll1524*-*Ptrc:gcy1p*-*kanR*	WT	No	No erythritol [36d–10.7]
SEP004	*slr0168::Ptrc:tm1254*-*Ptrc:alr1*-*kanR*	WT	Yes	No erythritol [36d–17.7]
SEP005	*slr0168::Ptrc:yidA*-*Ptrc:alr1*-*kanR*	WT	Yes	No erythritol [36d–15.6]
SEP006	*slr0168::Ptrc:sll1524*-*Ptrc:alr1*-*kanR*	WT	No	No erythritol [36d–17.6]
SEP007	*slr0168::Ptrc:tm1254*-*Ptrc:gld1*-*kanR*	WT	Yes	0.55 mM [36d–17.6]
SEP007m	*slr0168::Ptrc:tm1254*-*Ptrc:gld1*	WT	Yes	0.31 mM [30d–17.6]
SEP008	*slr0168::Ptrc:yidA*-*Ptrc:gld1*-*kanR*	WT	Yes	0.05 mM [23d–17.3]
SEP009	*slr0168::Ptrc:sll1524*-*Ptrc:gld1*-*kanR*	WT	No	No erythritol [28d–14.7]
SEP010	*slr0168::Ptrc:tm1254*-*Ptrc:pc20*-*kanR*	WT	Yes	0.04 mM [28d–22.6]
SEP011	*slr0168::Ptrc:yidA*-*Ptrc:pc20*-*kanR*	–	–	–
SEP012	*slr0168::Ptrc:sll1524*-*Ptrc:pc20*-*kanR*	WT	No	No erythritol [28d–24.4]
SEP013	*pAVO*-*Ptrc:tm1254*-*Ptrc:gld1*-*kanR*	WT	Plasmid	0.98 mM [30d–19.8]
SEP015	*slr0168::Ptrc:tm1254*-*Ptrc:gld1* *pVZ*-*Ptrc:tm1254*-*kanR*	SEP007 m	Plasmid	0.27 mM [30d–19.5]
SEP016	*slr0168::Ptrc:tm1254*-*Ptrc:gld1* *pAVO*-*PcpcBA:H*-*tm1254*-*specR*	SEP007 m	Plasmid	1.0 mM [33d–21.2]
SEP017	*slr0168::Ptrc:tm1254*-*Ptrc:gld1* *pAVO*-*PcpcBA:H*-*yidA*-*specR*	SEP007 m	Plasmid	0.65 mM [33d–22.1]
SEP021	*pAVO* + -*PcpcBA:H*-*tm1254*-*Ptrc:gld1*-*specR*	WT	Plasmid	1.45 mM [28d–17.6]
SEP022	*phaA::PpsbA2:tktS7002*-*His*-*camR* *pAVO* + -*PcpcBA:H*-*tm1254*-*Ptrc:gld1*-*specR*	SRP005	Plasmid	1.9 mM [28d–12.9]
SEP023	*phaA::Ptrc:tktS7002*-*His*-*camR* *pAVO* + -*PcpcBA:H*-*tm1254*-*Ptrc:gld1*-*specR*	SRP006	Plasmid	0.66 mM [28d–12.9]
SEP024	*phaA::PpsbA2:pktS7942*-*His*-*camR* *pAVO* + -*PcpcBA:H*-*tm1254*-*Ptrc:gld1*-*specR*	SRP007	Plasmid	2.1 mM [28d–13.6]
SEP025	*phaA::Ptrc:pktS7942*-*His*-*camR* *pAVO* + -*PcpcBA:H*-*tm1254*-*Ptrc:gld1*-*specR*	SRP008	Plasmid	1.7 mM [28d–15.0]
SRP005	*phaA::PpsbA2:tktS7002*-*His*-*camR*	WT	Yes	NA
SRP006	*phaA::Ptrc:tktS7002*-*His*-*camR*	WT	Yes	NA
SRP007	*phaA::PpsbA2:pktS7942*-*His*-*camR*	WT	Yes	NA
SRP008	*phaA::Ptrc:pktS7942*-*His*-*camR*	WT	Yes	NA

Strains are listed with their respective phosphatase, including *tm1254*, *yidA* or *sll1524*, and erythrose reductase, including *cmER, gcy1p, alr1, gld1* or *pc20* (P*trc1* and P*cpcBA* indicate the used promoters). *Slr0168* and *phaA* mark the respective genes at which genomic insertion took place, whereas pVZ and pAVO are replicative plasmids

*NA* not applicable. *kanR* kanamycin resistance cassette, *specR* spectinomycin resistance cassette, *camR* chloramphenicol resistance cassette

**Fig. 4:**
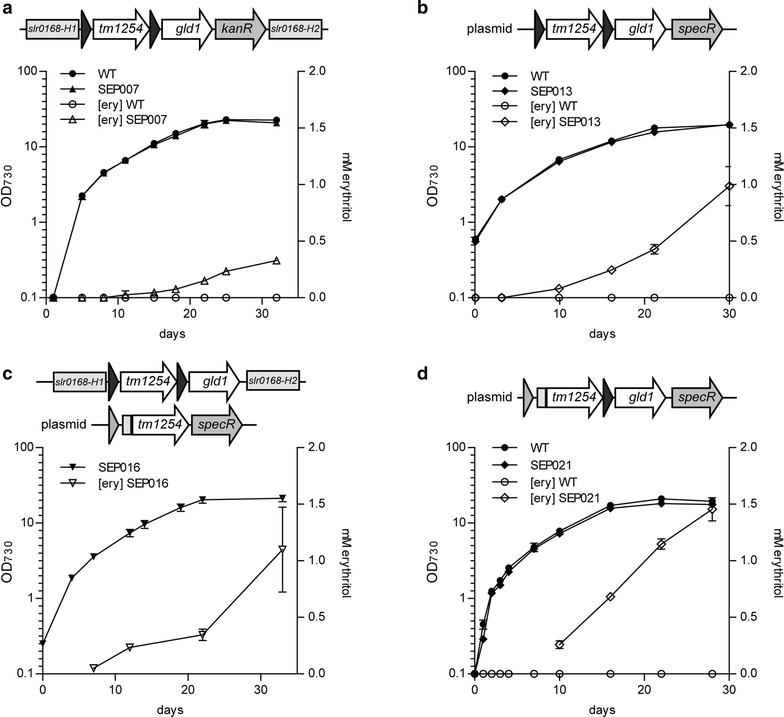
Cyanobacterial production of erythritol. Growth and extracellular erythritol production of wildtype *Synechocystis* (WT) and mutants SEP007 (**a**), SEP013 (**b**), SEP016 (**c**), SEP021 (**d**). *Solid symbols* represent OD_730_, whereas* open symbols* depict the erythritol concentration. *Error bars* represent SD (n = 2). Genetic representation shows the inserted genes for the mutants, represented as arrows (not to scale). The *light grey bar* in front of *tm1254* represent the decahistidine tag, whereas the *dark and light grey short arrows* represent the *trc1* and the *cpcBA* promoter, respectively

To assess the expression level and activity of the heterologous enzymes, we prepared soluble lysates of the different *Synechocystis* mutant strains. First, these lysates were analyzed using SDS-PAGE, combined with CBB staining (Fig. 3b). The proteins TM1254, ErCm and Gcy1p were also separately overexpressed in *E. coli* to confirm the expected position of each protein on the gel. No detectable differences in expressed protein between the lysates of wild type *Synechocystis* and the mutant strains SVPV2, SVPV3 and SAW029 were observed, indicating that expression of the heterologous enzymes does not lead to amounts that can be visualized by direct (protein) staining. In SAW030, however, we did observe an additional band corresponding to the molecular weight of the erythrose reductase Gcy1p (Fig. 3b). No stained band was visible for the TM1254 phosphatase. This might in part be due to the relatively high protein abundance at the molecular weight range in *Synechocystis* lysates where TM1254 is to be expected (Fig. 4). Moreover, it has been observed earlier that a heterologous enzyme in *Synechocystis* could not be detected by visual inspection of SDS-PAGE gels, although it did show significant enzymatic activity [17].

Next, activity of the erythrose reductases was measured by monitoring consumption of NADPH at pH 7.6 in soluble lysates, upon addition of D-erythrose. As anticipated on basis of the results obtained with SDS-PAGE, we did not detect any erythrose reductase activity for mutant strains SVPV2, SVPV3 and SAW029. However, in SAW030 we did observe activity for Gcy1p, in the order of ~0.05 mmol/gDW/h, which was just above the detection level of the assay (Additional file 1: Figure S1).

The relatively low reductase activity observed in this strain indicates that activity of Gcy1p in SVPV3 could be below detection levels, as the expression level of a chromosomally integrated construct tends to be roughly 3 to 4 times lower than when the same gene is expressed from an RSF1010-derived plasmid [28].

### Testing additional enzymes for erythritol production

As the next step we tested novel enzymes, based on their more favorable catalytic properties, which are described in Additional file 1: Tables S3, S4. These enzymes included several erythrose reductases of fungal origin, namely ALR1, GLD1, and Pc20g15580, derived from *Aspergillus niger, Hypocrea jecorina* and *Penicilium chrysogenum,* respectively [12]. We only found one additional erythrose-4-phosphate-specific phosphatase in the literature, i.e. YidA, derived from *E. coli* [29]. We therefore selected this enzyme, as well as an endogenous enzyme from *Synechocystis*, Sll1254, that showed the highest sequence similarity to YidA. The genes encoding erythrose reductases as well as the novel phosphatase YidA were synthesized, after codon optimization for *Synechocystis*, behind a *trc1* promoter and provided with a transcriptional terminator. The (presumed) phosphatase *sll1524* was amplified directly from *Synechocystis*. To test for the best combination of enzymes, we made 12 different combinations of one phosphatase and one reductase, each with their own *trc1* promoter (Table 1). These gene-pairs were cloned into a pHeKHe vector that can be used for genomic integration into the *Synechocystis* genome at *sll0168*. pHeKHe is a derivative of pHKH001 [16] that was adjusted with extended homologous regions to facilitate, and increase the frequency of, genomic integration. Only the combination of YidA and Pc20g15580 (pEP011) failed in the cloning phase, most likely due to toxicity effects in *E. coli* and was therefore not tested. All other vectors were successfully integrated into the *Synechocystis* genome, but none of the constructs containing the *sll1524* gene could be fully segregated (Table 1). The resulting strains, named SEP001 to SEP012, were tested for erythritol production in a moderate light intensity (35 μE/m^2^/s white light) in BG-11 medium supplemented with 25 mM CAPSO (pH = 9). Moreover, activity of the erythrose reductases was measured in soluble lysates derived from these cultures, after addition of NADPH and d-erythrose. The results are depicted in Table 1.

**Fig. 5:**
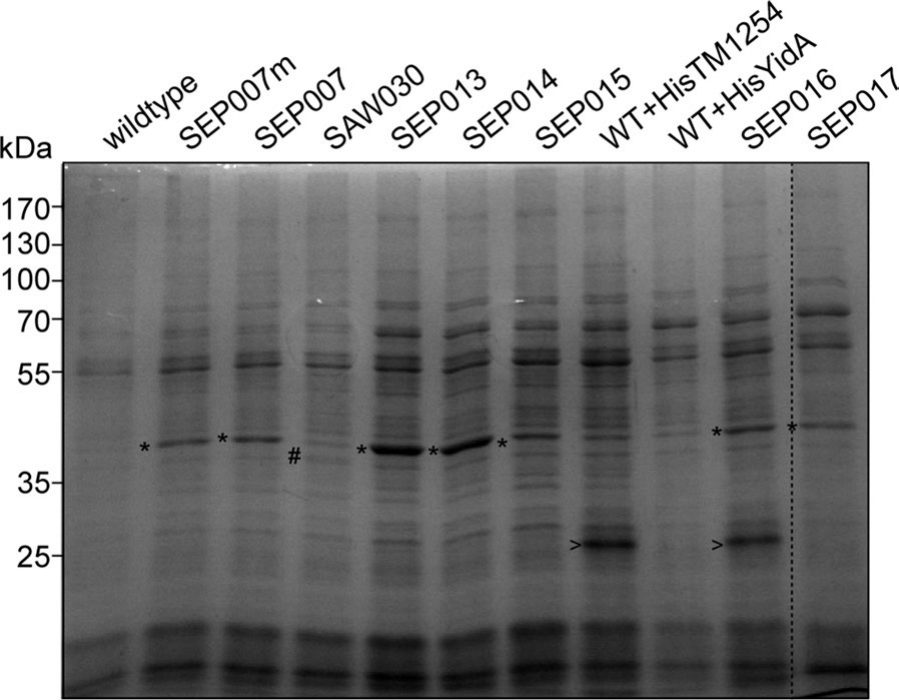
CBB-stained SDS-PAGE analysis of *Synechocystis* cell lysates. Soluble lysates of wildtype and relevant mutant strains. Protein bands are indicated with * GLD1, # Gcy1p, > His-TM1254, respectively

No erythritol production was observed for the strains (over)expressing the endogenous phosphatase Sll1524, nor for the strains with the erythrose reductase ALR1. The latter can likely be explained by the lack of protein expression, observed on SDS-PAGE for this enzyme. The Sll1524-expressing strains did not fully segregate, indicating toxicity of the expression of the heterologous genes, and this might be the reason that no erythritol production was observed. The other strains did show some erythritol formation, with SEP007 showing the highest titers, which indicates that TM1254 and GLD1 is the best combination of enzymes (Table 1; Fig. 4a). In contrast to SAW030, this strain showed no simultaneous production of glycerol. Furthermore, the GLD1 reductase showed significantly higher protein expression and enzyme activity than was observed previously for Gcy1p of SAW030 (Fig. 5 and Additional file 1: Figure S1), even though the latter enzyme was expressed from a (multicopy) plasmid. Interestingly, Pc20g15580 shows comparable activity levels as GLD1, but does not result in significant erythritol production (Table 1). Although YidA is expected to be the best phosphatase, based on its catalytic properties (Additional file: 1 Table S4), the use of this phosphatase does not lead to higher erythritol levels than with the use of TM1254. Therefore, from here onwards we focused fully on the use of the TM1254 phosphatase.

### Increasing phosphatase and reductase expression levels

To increase erythritol production of our mutant strains by higher expression of the preferred enzymes, a conjugative plasmid with TM1254 and GLD1, each with their own *trc1* promoter, (*pAVO*-*tm1254*-*gld1*) was constructed and introduced into wildtype *Synechocystis,* which resulted in strain SEP013. This strain should have phosphatase expression levels that are comparable to the SAW030 strain, and, with the better reductase, might produce more erythritol than SAW030. Although the higher expression level of the reductase was confirmed by SDS-PAGE (Fig. 5) and activity assays (Additional file 1: Figure S1), the levels of erythritol production were not increased by much (Fig. 4b), as compared to SAW030. Next, we tried to specifically increase phosphatase levels. To achieve this, we first re-created strain SEP007, but now without the kanamycin resistance marker, to allow for additional genetic modifications. This strain, SEP007m, showed similar reductase activity and erythritol production as SEP007. Next, we introduced the vector *pVZ*-*TM1254* into SEP007m and tested for erythritol production. However, this strain (i.e. SEP015) did not produce more erythritol than SEP007m (Table 1).

Since the phosphatase enzyme in the mutant strains could not be detected on a CBB-stained SDS-PAGE, nor measured for its enzymatic activity, we decided to reclone TM1254 and YidA with an N-terminal deca-histidine tag under the control of the *cpcBA* promoter into a conjugative plasmid. These plasmids, *pAVO*-*cTM125* and *pAVO*-*cYidA,* were introduced into wildtype *Synechocystis* as well as into SEP007m, the latter resulting in strains SEP016 and SEP017, respectively. Interestingly, the N-terminal deca-histidine tag seemed to have a major effect on the TM1254 protein expression level, but not on that of YidA (Fig. 5). This was confirmed by Western blotting and staining with an anti-poly-histidine antiserum (not shown). The new N terminus may have positively affected mRNA stability and/or initiation of translation of TM1254. Next, SEP016 and SEP017 were tested for erythritol production. Whereas erythritol production levels for SEP017 were not much higher than observed for SEP007 (Table 1), SEP016 was clearly producing more erythritol (Fig. 4c). This shows that increased expression of TM1254 does lead to higher erythritol production, although the final levels did not differ too much from those in SEP013.

As a final optimization step, the approaches used to construct SEP013 and SEP016 were combined by putting both the *cpcBA*-driven poly-His tagged *tm1254* and *trc1:gld1* in a single conjugation vector (pEP021). Introducing this vector into *Synechocystis* resulted in strain SEP021. In a growth- and production experiment, SEP021 showed erythritol production up to 1.45 mM in 28 days (Fig. 4d). Looking at protein expression levels on a CBB-stained SDS-PAGE, it was noted that GLD1 expression in SEP021 is comparable to SEP013. The TM1254 expression of SEP021 is higher than in SEP013, but seems to be lower than was observed for SEP016 (compare Fig. 5 and Additional file 1: Figure S2). Whether the expression levels of TM1254 from the plasmid are negatively affected by the high expression of GLD1 or whether TM1254 displays some level of instability is at this point unclear. The difference in erythritol titers between SEP013 and SEP021 indicates that TM1254 expression was clearly limiting erythritol production in SEP013. Although TM1254 expression is raised in SEP021, it might still be limiting and/or its off-target activity becomes dominant. In conclusion, the expression levels of both the GLD1 erythrose reductase and TM1254 phosphatase were significantly raised. However, the effect on erythritol production levels were marginal.

### No significant intracellular accumulation of erythritol

To explore whether the relatively low extracellular erythritol production levels were due to intracellular accumulation of this metabolite, its internal concentration was also analyzed. For this reason, samples of SEP021 were prepared as in [24], and the concentration of intracellular metabolites was analyzed using the HPLC method. After recalculating the measured concentration back to the cell volume (see “Methods”), we found that the internal concentration of erythritol would be in the range of 1–2 mM. This indicates that there is no significant accumulation of intracellular erythritol in our production strain and that the cytoplasmic membrane only marginally—if at all functions as a diffusion barrier to this metabolite.

### Increasing flux towards the precursor E4P

**Fig. 6:**
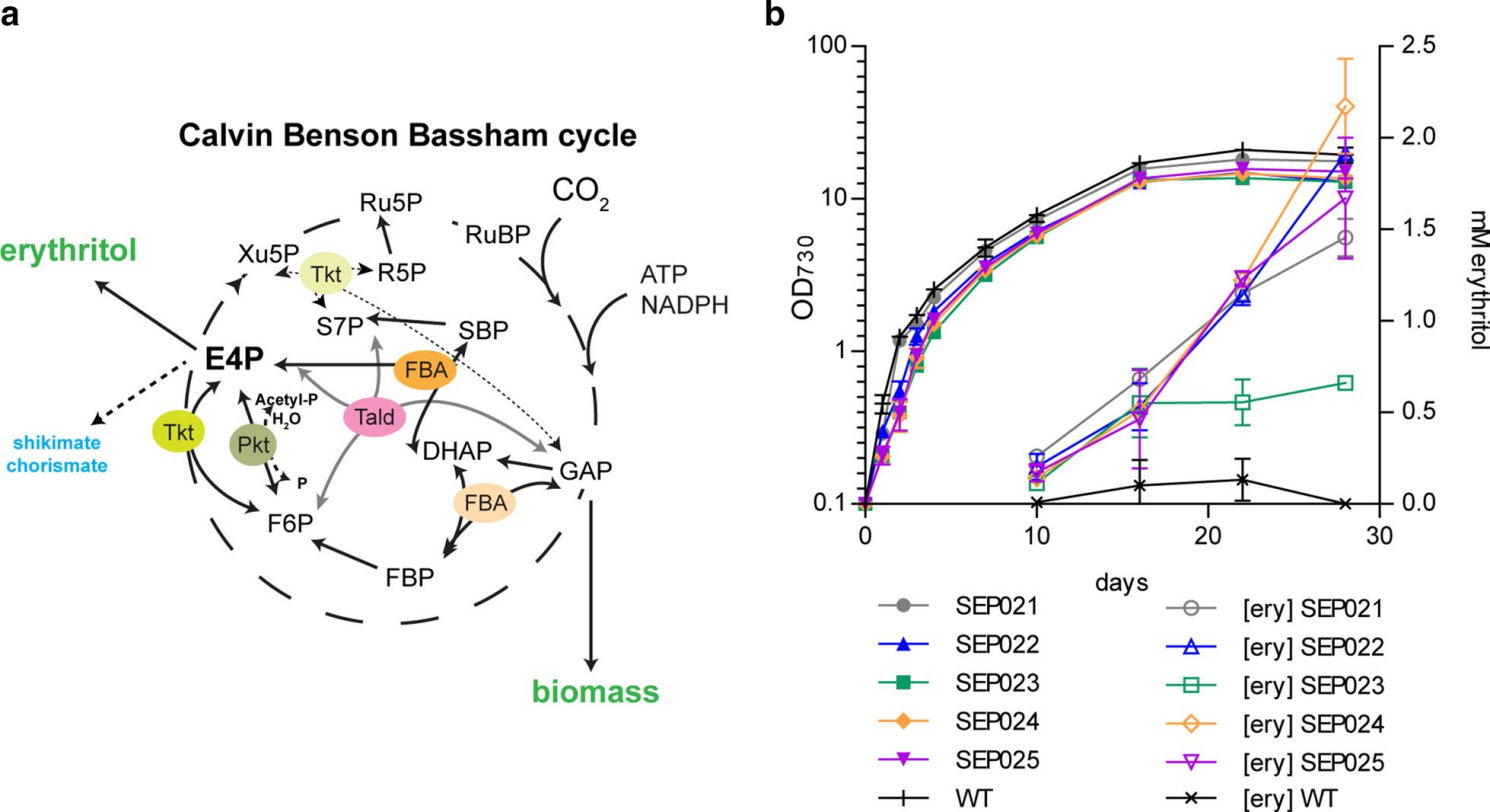
Increasing flux towards erythrose-4-phosphate. **a** Schematic representation of the CBB cycle intermediates and the reactions/enzymes affecting the E4P concentration. Abbreviations: *Tkt* transketolase, *Pkt* phosphoketolase, *Tald* transaldolase, *FBA* fructose-1,6-bisphosphate aldolase, *GAP* glyceraldehyde-3-phosphate, *F6P* fructose-6-phosphate, *FBP* fructose-1,6-bisphosphate, *Xu5P* xylulose-5-phosphate, *S7P* seduheptolose-7-phosphate, *SBP* sedoheptulose-1,7-bisphosphate, *R5P* ribose-5-phosphate, *Ru5P* ribulose-5-phosphate, *RuBP* ribulose-1,5-bisphosphate. **b** Growth and extracellular erythritol production of wildtype *Synechocystis* (WT) and mutants SEP021 to SEP025. Cells were grown under moderate = intensity illumination (~30 μE/m^2^/s) in BG-11, supplemented with 25 mM CAPSO (pH = 9) at 30 °C. *Solid symbols* represent OD_730_, whereas *open symbols* depict the erythritol concentration. *Error bars* represent SD (n = 2)

Further optimization work on our erythritol producing strain was dedicated to increasing the availability of the precursor metabolite E4P. There are multiple ways to increase the flux towards E4P, e.g. by modification of different parts of the Calvin cycle. Four different reactions affect the intracellular E4P concentration directly (Fig. 6a). First, the transketolase (Tkt) can convert glyceraldehyde-3-phosphate (GAP) with fructose-6-phosphate (F6P) into E4P and xylulose-5-phosphate (Xu5P). Secondly, the phosphoketolase (Pkt) can convert F6P with phosphate into E4P and Acetyl phosphate. Thirdly, transaldolase (Tald) can convert GAP and seduheptolose-7-phosphate (S7P) into E4P and F6P and, lastly, Fructose-1,6-bisphosphate aldolase (FBA) catalyzes the reversible conversion of FBP into GAP and DHAP. This latter enzyme also has affinity for S7P, which then is cleaved into DHAP and E4P. There are two classes of aldolases: class I FBA (CI-FBA) does not require divalent cations for their activity, whereas class II FBA (CII-FBA) does (and is inhibited by EDTA). Interestingly, the *Synechocystis* genome encodes both a CI-FBA (*slr0943*) and a CII-FBA (*sll0018*), but the CII-FBA accounts for 90 % of the cellular FBA activity. The gene encoding CI-FBA can be disrupted and therefore this enzyme seems to be functionally redundant, whereas attempts of marker insertion into CII-FBA failed [30]. Under photoautotrophic conditions, it seems likely that Tkt and Pkt will contribute to increased E4P production, whereas Tald and FBA are assumed to contribute to E4P consumption. Since it has been shown that CII-FBA cannot be disrupted [30], and the same presumably holds for Tald, we aimed at overexpression of Tkt and Pkt.

Using a fusion PCR method, the *tkt* and *pkt* genes extended with a C-terminal histidine tag were inserted into the *phaA* insertion site behind a t*rc1* or a *psbA2* promoter, together with a chloramphenicol resistance cassette. To avoid unwanted genomic rearrangements, not the endogenous genes were chosen, but rather those from the related species *Synechococcus*. The resulting strains SRP005, SRP006, SRP007 and SRP008 were conjugated with the pEP021 plasmid to create SEP022-SEP025, respectively.

For these strains we isolated cell free extracts to analyze the expression level of the heterologously expressed proteins on a CBB-stained SDS-PAGE (Additional file 1: Figure S2). The Tkt and Pkt were highly expressed behind the *trc1* promoter, but not visible with the *psbA2* promoter (confirmed by Western blotting). Moreover, all strains showed high-level expression of the GLD1 protein and reasonable levels of TM1254, comparable to the SEP021 strain. These strains were also tested in a growth and production experiment, but the erythritol levels were quite similar to SEP021 up until day 22 (Fig. 6b). One of the strains produced significantly less erythritol, namely the Tkt-overexpression strain SEP023. Perhaps, the high expression of *tkt* behind the *Ptrc* promoter has a negative effect on production. We did observe that all the Tkt and Pkt overexpression strains had a slightly reduced growth rate and final optical density (Table 1). Interestingly, the final erythritol titers on day 28 for SEP022, 24 and 25 were slightly higher than for SEP021, with a maximum of 2.1 mM produced in SEP024. However, the large standard deviation for titers at this timepoint do not allow a significant conclusion for a positive or negative effect of the respective Pkt or Tkt overexpression. These results indicate that the heterologous enzymes for erythritol production are likely still fully controlling the reaction here.

## Conclusions

In this study, we have shown proof of principle for photosynthesis- and CO_2_-based production of erythritol in engineered cyanobacteria. Highest product titers were ~2.1 mM after culturing for 28 days. To our knowledge, this is the first reported production of erythritol in any genetically engineered (micro)organism that does not naturally produce erythritol. The erythritol titers achieved are still quite low, however, even for cyanobacterial production systems. The reason for this limited production is unknown. Moreover, product formation is only observed when the culture is close to/has reached stationary phase. This observation is comparable to what is observed in the production of another polyol, mannitol, in cyanobacteria [31]. Jacobsen and Frigaard showed that the extracellular concentration of mannitol started to increase (linearly as a function of time) only after the producing cells had exited the exponential phase. However, for mannitol it was observed that there was significant intracellular accumulation, which we did not detect in our strains for erythritol.

In one of the first production strains, SAW030, there was considerable co-production of glycerol next to the produced erythritol, which was not observed in a strain with lower expression levels of the same enzymes (i.e. SEP001). It has been shown that the production of glycerol can be accomplished by introduction of a dedicated glycerol-3-phosphate phosphatase [32]. However, no affinity for glycerol-3-phosphate has been described for the used phosphatase TM1254 [27]. Also, none of the other strains with high phosphatase expression exhibited glycerol production to the same extent as SAW030, although some glycerol was observed occasionally. It therefore seems likely that the glycerol production is primarily due to the high expression of Gcy1p, which has also been described to have affinity for d-glyceraldehyde [10]. The intracellular abundance of glyceraldehyde is not known, but glyceraldehyde-3-P is an important precursor for terpenes and sterols and likely more abundant than E4P. It might thus also be that both the phosphatase and reductase are used for the glycerol production.

The availability of E4P could also present a problem for higher levels of product formation. There is a limited amount of data available on the absolute concentration of metabolites in *Synechocystis* and these do not include E4P [13, 33]. The precursor of mannitol, F6P, is used for production of glycogen, exopolysaccharides and various sugars, which jointly can constitute a major component of the cells under certain growth conditions [34, 35]. In contrast, E4P only serves as a precursor for the shikimate/chorismate pathway and is therefore likely not a highly abundant metabolite in exponentially growing cells. It seems likely that the flux towards this metabolite is not as large as to pyruvate or F6P, which are major tapping points for product formation from CO_2_ in cyanobacteria [8]. We were expecting to improve erythritol production levels by increasing E4P availability via overexpression of a phosphoketolase or transketolase. However, the resulting effects were minimal.

The latter observation is to be expected if the two heterologous enzymes involved in erythritol production are still fully limiting/rate-controlling the production pathway. If so, this finding would then be fully in line with previous results obtained with the production of l-lactic acid, where we showed that increasing the availability of the precursor molecule (pyruvate) only had an effect when the heterologous product-forming enzyme (lactate dehydrogenase in that case) was no longer fully controlling the rate of the reaction of product formation [28]. In this study, we have shown that the expression levels of the erythrose reductase as well as of the phosphatase were considerable, to the extent that they are visible on a CBB-stained SDS-PAGE. However, whereas we could find considerable activity levels for the reductase, we were unable to test separately for phosphatase activity. Moreover, available literature on the phosphatase used, TM1254, indicates that this particular enzyme is not fully specific for E4P and also that its catalytic properties are not optimal [27]. Therefore, we expect that in the future, production levels may be further improved by optimizing the phosphatase used in those studies.

## Additional file

10.1186/s12934-016-0458-y **Figure S1**. Erythrose reductase activity of Synechocystis soluble lysates; **Figure S2**. CBB-stained SDS-PAGE analysis of the soluble lysates of mutants SEP021 to SEP025; **Table S1**. Primers used in this study; **Table S2**. E. coli strains and plasmids used in this study; **Table S3**. Erythrose reductases and their published catalytic characteristics; **Table S4**. Erythrose-4-phosphatases and their published catalytic characteristics.

## Abbreviations

CBB: coomassie brilliant blue
E4P: d-erythrose-4-phosphate
F6P: fructose-6-phosphate
FBA: fructose-1,6-bisphosphate aldolase
GAP: glyceraldehyde-3-phosphate
LB: lysogeny broth
Pkt: phosphoketolase
S7P: seduheptolose-7-phosphate
SD: standard deviation
Tald: transaldolase
Tkt: transketolase
WT: wild type
Xu5P: xylulose-5-phosphate
