# Supplementary material for: Genetic engineering of Synechocystis PCC6803 for the photoautotrophic production of the sweetener erythritol
Source: Microb Cell Fact. 2016 Apr 8;15:60. doi: 10.1186/s12934-016-0458-y (PMC4826498; doi:10.1186/s12934-016-0458-y)
Supplement: Supplementary file 1 — 10.1186/s12934-016-0458-y Figure S1. Erythrose reductase activity of Synechocystis soluble lysates; Figure S2. CBB-stained SDS-PAGE analysis of the soluble lysates of mutants SEP021 to SEP025; Table S1. Primers used in this study; Table S2. E. coli strains and plasmids used in this study; Table S3. Erythrose reductases and their published catalytic characteristics; Table S4. Erythrose-4-phosphatases and their published catalytic characteristics. [file 12934_2016_458_MOESM1_ESM.pdf]

SUPPLEMENTAL DATA
Genetic engineering of *Synechocystis* PCC6803 for the photoautotrophic production of the sweetener erythritol

Aniek D. van der Woude[1*], Ruth Perez Gallego[1], Angie Vreugdenhil[1], Vinod Puthan Veetil[1], Tania Chroumpi[1], Klaas J. Hellingwerf[1]

Supplemental Figure S1

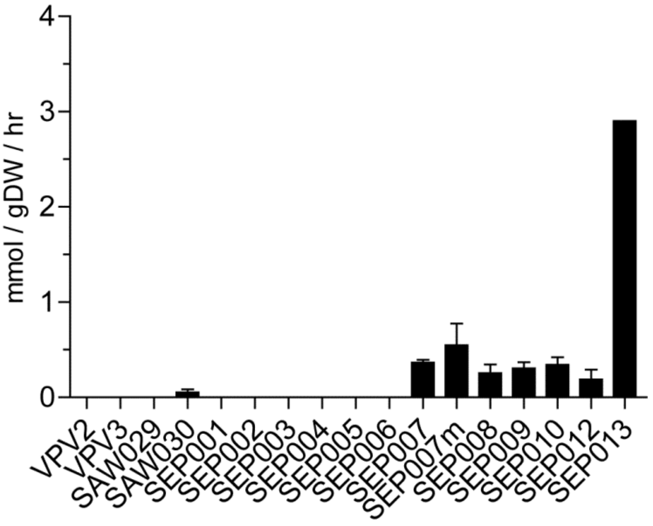

Figure S1. Erythrose reductase activity of *Synechocystis* soluble lysates. Enzymatic activity of ER in soluble lysates of relevant mutants, using NADPH as the co-factor. Error bars represent the SD of biological replicates (n=3).

Supplemental Figure S2

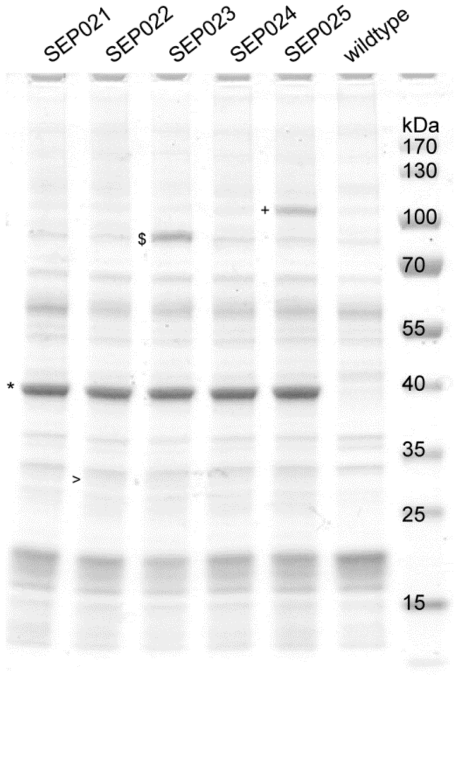

Figure S2. CBB-stained SDS-PAGE analysis of the soluble lysates of mutants SEP021 to SEP025. Overexpressed proteins are indicated as follows: * GLD1, > His-TM1254, $ Tkt-His, + Pkt-His.

Supplemental table 1. Primers used in this study

**Name**	**Sequence***	**Additional Information**
**H1seq_F**	AATGGTCCCAAAATTGTC	Colony PCR segregation at *slr0168*
**H2seq_R**	CTGTGGGTAGTAAACTGGC	Colony PCR segregation at *slr0168*
**KanF_back_Seq**	TCCCGTTGAATATGGCTC	Colony PCR *slr0168* insertion
**HR1b_F**	CCCAAACTAAACCCGATGAC	Colony PCR segregation at *phaA*
**HR2b_R**	CCCCCATTTCCTGGAGTAAT	Colony PCR segregation at *phaA*
**CAM-out-F**	ATAAGCGGATGAATGGCAGA	Colony PCR *phaA* insertion
**psbA2_H1seqF**	GCCAACATCGGTTTTGAAGT	Colony PCR segregation at *psbA2* insertion
**Psb_seq_R**	CCATAATTCCTCTATCGAAGATGGA	Colony PCR segregation at *psbA2* insertion
**SpecR_out_F**	CCGAGGCATAGACTGTACCC	Colony PCR insertion pDF / pAVO
**TM_BamH1_F**	ccatcacggatccGAGGCTGTCATCTTCGATATGG	cloning TM1254 in pQE30
**TM_Hind3_R**	ctagtaaagcttATCACAACACTTCTTTAAGCACGTTC	cloning TM1254 in pQE30
**ER_BamH1_F**	ccatcacggatccTCCAGCACTTACACTCTCACC	cloning ErCm in pQE30
**ER_Hind3_R**	ctagtaaagcttATCAACGCCGCGCAAGGGCGTCGATAC	cloning ErCm in pQE30
**GcY_BamH1_F**	ccatcacggatccCCTGCTACTTTGCACGACTCCAC	cloning Gcy1p in pQE30
**GcY_Hind3_R**	ctagtaaagcttATCATTTGAATACTTCAAAAGGGGACC	cloning Gcy1p in pQE30
**JBS315**	GATGTATGCTCTTCTGCTC	Colony PCR insertion pVZ/pDF/pAVO
**JBS316**	CTGCCCGGATTACAGATC	Colony PCR insertion pVZ/pDF/pAVO
**MluI_pDFOmega_F**	aaa*ACGCGT*caagcgagctcgatatccgt	Replace kan with omega in pVZ
**MluI_pDFOmega_R**	aaa*ACGCGT*atgccgggagtgtacaaagg	Replace kan with omega in pVZ
**sll1524_F**	AACGAGTGTCAATCAAGTTAGTAGT	Amplify *sll1524*
**sll1524_R**	tccTTAACAGAGTGCTAGGGTTG	Amplify *sll1524*
**H2ext_R_KpnI**	AAATGGTACCCGCCCCATCTTACCTGAATA	extending homologous regions *slr0168*
**H1ext_F_SacI**	AAATGAGCTCCCAGTAAGGTCACCCATCGT	extending homologous regions *slr0168*
**H1_R_SacII**	AAAACCGCGGCCATATAAATCCCCGCCACT	extending homologous regions *slr0168*
**H2_F_Xho**	AAATCTCGAGAGACCAAGCCCAATTTCGTTTG	extending homologous regions *slr0168*
**pAVO_mobaY25F_F**	CAAGGCCGACTTCATCCAGCGCGAAG	introducing Y25F mutation in MobA
**pAVO_mobaY25F_R**	CTTCGCGCTGGATGAAGTCGGCCTTG	introducing Y25F mutation in MobA
**SpecRend_F**	GGCGAGATCACCAAGGTAGTCGGC	introducing Y25F mutation in MobA
**RepB_R**	CTACATGCTGAAATCTGGCCCGCCC	introducing Y25F mutation in MobA
**Tkt-H1-R**	gagacagtactaggtCATCACCATCACCATCAC	Tkt overexpression mutant
**H1-Tkt-F**	atggtgatggtgatgACCTAGTACTGTCTCAGCG	Tkt overexpression mutant
**H2-PpsbA2-Tkt-R**	ataggaggacctaggATGACCGTTGCAACCCAATC	Tkt overexpression mutant
**Tkt-PpsbA2-H2-F**	ggttgcaacggtcatCCTAGGTCCTCCTATGTATTTG	Tkt overexpression mutant
**H2-Ptrc-tkt-R**	aaagaggagaaatgacatATGACCGTTGCAACCCAATC	Tkt overexpression mutant
**tkt-Ptrc-H2-F**	ggttgcaacggtcatATGTCATTTCTCCTCTTTAATG	Tkt overexpression mutant
**Pkt-H1-R**	tggcaatggcctttcCATCACCATCACCATCAC	Pkt overexpression mutant
**H1-Pkt-F**	atggtgatggtgatgGAAAGGCCATTGCCACTG	Pkt overexpression mutant
**H2-PpsbA2-Pkt-R**	ataggaggacctaggATGACAAGTACCTTGCAAG	Pkt overexpression mutant
**Pkt-PpsbA2-H2-F**	caaggtacttgtcatCCTAGGTCCTCCTATGTATTTG	Pkt overexpression mutant
**H2-Ptrc-Pkt-R**	aaagaggagaaatgacatATGACAAGTACCTTGCAAG	Pkt overexpression mutant
**Pkt-Ptrc-H2-F**	caaggtacttgtcatATGTCATTTCTCCTCTTTAATG	Pkt overexpression mutant

Table S2. *E. coli* strains and plasmids used in this study.

Strain/plasmid	Precursor	*	Relevant Insert(s) or resulting genotype**	Application	Reference
E. coli XL-1 blue		-	TetR	Standard cloning host	Stratagene
E. coli EPI400		-		CopyCutterTM cells for cloning	Epicentre
pHKH001	pBluescript II SK+	-	Δslr0168::KanR, AmpR	Insertion at slr0168	(Angermayr et al., 2012)
pVPV1	pHKH001	S	P_{trc1}:Gcy1p_KanR	cloning pVPV3	this study
pVPV2	pHKH001	S	P_{trc1}: TM1254-ERCm_KanR	strain SVPV2	this study
pVPV3		C	P_{trc1}: TM1254-Gcy1p_KanR	strain SVPV3	this study
pJBS1250	RSF1010 derivative	-	kanR	cloning pVZ-TM1254	(Chen et al, submitted)
pVZ-TM1254	pVZ321	C	pVZ_P_{trc1}: TM1254_KanR	cloning + strain SEP015	this study
pAW029	pVZ-TM1254	C	pVZ_P_{trc1}: TM1254-P_{trc1}:ERCm_KanR	strain SAW029	this study
pAW030	pVZ-TM1254	C	pVZ_P_{trc1}: TM1254-P_{trc1}:Gcy1p_KanR	strain SAW030	this study
pQE30		-	N-His_AmpR		Qiagen
pQE30-TM1254	pQE30	C	TM1254_AmpR	expression in *E. coli*	this study
pQE30-Gcy1p	pQE30	C	Gcy1p_AmpR	expression in *E. coli*	this study
pQE30-ErCm	pQE30	C	ERCm_AmpR	expression in *E. coli*	this study
pUC57-coALR1	pUC57	S	P_{trc1}:ALR1_AmpR	cloning pEP004-pEP006	this study
pUC57-coGLD1	pUC57	S	P_{trc1}:GLD1_AmpR	cloning pEP007-pEP009	this study
pUC57-coYidA	pUC57	S	P_{trc1}:YidA_AmpR	cloning	this study
pCCI_coPc20g15580	pCCI	S	P_{trc1}:Pc20g15580_AmpR	cloning pEP010-pEP012	this study
pHKH_sll1524	pHKH001	C	P_{trc1}:sll1524_KanR	cloning	this study
pHeKHe_sll1524	pHKH_sll1524	C	P_{trc1}:sll1524_KanR	cloning pEP003, pEP006, pEP009, pEP012	this study
pHeKHe_TM1254	pHeKHe_sll1524	C	P_{trc1}: TM1254_KanR	cloning pEP001, pEP004, pEP007, pEP010	this study
pHeKHe_YidA	pHeKHe_sll1524	C	P_{trc1}:YidA_KanR	cloning pEP002,pEP005,pEP008	this study
pEP001	pHeKHe_TM1254	C	P_{trc1}:TM1254_P_{trc1}:Gcy1p_KanR	strain SEP001	this study

pEP002	pHeKHe_YidA	C	P_{trc1}:YidA_P_{trc1}:Gcy1p_KanR	strain SEP002	this study
pEP003	pHeKHe_sll1524	C	P_{trc1}:sll1524_P_{trc1}:Gcy1p_KanR	strain SEP003	this study
pEP004	pHeKHe_TM1254	C	P_{trc1}:TM1254_P_{trc1}:ALR1_KanR	strain SEP004	this study
pEP005	pHeKHe_YidA	C	P_{trc1}:YidA_P_{trc1}:ALR1_KanR	strain SEP005	this study
pEP006	pHeKHe_sll1524	C	P_{trc1}:sll1524_P_{trc1}:ALR1_KanR	strain SEP006	this study
pEP007	pHeKHe_TM1254	C	P_{trc1}:TM1254_P_{trc1}:GLD1Hj_KanR	strain SEP007	this study
pEP007m	pEP007	C	P_{trc1}:TM1254_P_{trc1}:GLD1Hj	strain SEP007m	this study
pEP008	pHeKHe_YidA	C	P_{trc1}:YidA_P_{trc1}:GLD1Hj_KanR	strain SEP008	this study
pEP009	pHeKHe_sll1524	C	P_{trc1}:sll1524_P_{trc1}:GLD1Hj_KanR	strain SEP009	this study
pEP010	pHeKHe_TM1254	C	P_{trc1}:TM1254_P_{trc1}:Pc20g15580_KanR	strain SEP010	this study
pEP012	pHeKHe_sll1524	C	P_{trc1}:sll1524_P_{trc1}:Pc20g15580_KanR	strain SEP012	this study
pDF-tm1254-gld1	pDF-lac	C	pVZ_P_{trc1}: TM1254-P_{trc1}:GLD1_KanR	strain SEP013	this study
pDF-lac	RSF1010 derivative	-	specR	cloning pAVO+	(Guerrero et al., 2012)
pAVO-cYFP		C		cloning	this study
pAVO-cTM1254	pAVO-cYFP	C	P_{cpcBA}:His-TM1254	strain SEP016	this study
pAVO-cYidA	pAVO-cYFP	C	P_{cpcBA}:His-YidA	strain SEP017	this study
pAVO+-cTM1254	pAVO-cTM1254	C	pAVO+-P_{cpcBA}:His-TM1254	cloning	this study
pEP021	pAVO+-cTM1254	C	pAVO+-P_{cpcBA}:His-TM1254_$P_{trc1:}$GLD1	strains SEP021-SEP025	this study

* S: directly synthesized and sub-cloned at Genscript (NJ, USA), C: derived from cloning procedures, -: previously described, see reference.

Supplemental table 3. Erythrose reductases and their published catalytic characteristics.

		D-erythrose		NADPH		
**gene**	**donor organism**	**Km (mM)**	**Kcat (s^{-1})**	**Km**	**Kcat (s^{-1})**	**Ref**
ErCm	*Candida magnolia JH110*	8,5	7,6	0,016	48	(Lee et al., 2010)
Gcy1p	*Saccharomyces cerevisiae*	3,4	-	-	-	(Ookura and Kasumi, 2007)
GLD1	*Hypocrea jecorina (Trichoderma reesei)*	0,016-0,134	530-36,5	-	-	(Jovanović et al., 2013)
ALR1	*Aspergillus niger*	0,139	25			(Jovanović et al., 2013)
Pc20g15580	*Penicilium chrysogenum*	?	?			NA

Supplemental table 4. Erythrose-4-phosphatases and their published catalytic characteristics.

		D-erythrose-4-P			
**gene**	**donor organism**	**Km (mM)**	**Kcat (s^{-1})**	**Vmax (umol/min/ mg protein)**	**ref**
TM1254	*Thermotoga maritima MSB8*	0,152	-	2,63	(Kuznetsova et al., 2005)
YidA	*Escherichia coli*	0,019	19	-	(Kuznetsova et al., 2006)
sll1524	*Synechocystis PCC6803*	-	-	-	NA

References

Angermayr, S.A., Paszota, M., Hellingwerf, K.J., 2012. Engineering a cyanobacterial cell factory for production of lactic acid. Appl. Environ. Microbiol. 78, 7098–7106. doi:10.1128/AEM.01587-12

Guerrero, F., Carbonell, V., Cossu, M., Correddu, D., Jones, P.R., 2012. Ethylene synthesis and regulated expression of recombinant protein in Synechocystis sp. PCC 6803. PloS One 7, e50470. doi:10.1371/journal.pone.0050470

Jovanović, B., Mach, R.L., Mach-Aigner, A.R., 2013. Characterization of erythrose reductases from filamentous fungi. AMB Express 3, 43. doi:10.1186/2191-0855-3-43

Kuznetsova, E., Proudfoot, M., Gonzalez, C.F., Brown, G., Omelchenko, M.V., Borozan, I., Carmel, L., Wolf, Y.I., Mori, H., Savchenko, A.V., Arrowsmith, C.H., Koonin, E.V., Edwards, A.M., Yakunin, A.F., 2006. Genome-wide analysis of substrate specificities of the Escherichia coli haloacid dehalogenase-like phosphatase family. J. Biol. Chem. 281, 36149–36161. doi:10.1074/jbc.M605449200

Kuznetsova, E., Proudfoot, M., Sanders, S.A., Reinking, J., Savchenko, A., Arrowsmith, C.H., Edwards, A.M., Yakunin, A.F., 2005. Enzyme genomics: Application of general enzymatic screens to discover new enzymes. FEMS Microbiol. Rev. 29, 263–279. doi:10.1016/j.femsre.2004.12.006

Lee, D.-H., Lee, Y.-J., Ryu, Y.-W., Seo, J.-H., 2010. Molecular cloning and biochemical characterization of a novel erythrose reductase from Candida magnoliae JH110. Microb. Cell Factories 9, 43. doi:10.1186/1475-2859-9-43

Ookura, T., Kasumi, T., 2007. Yeast Gcy1p Reduces Erythrose and Erythrose-4-phosphate. Rep Natl Food Res Inst 71, 57－60.
